# Prospective Image Quality and Lesion Assessment in the Setting of MR-Guided Radiation Therapy of Prostate Cancer on an MR-Linac at 1.5 T: A Comparison to a Standard 3 T MRI

**DOI:** 10.3390/cancers13071533

**Published:** 2021-03-26

**Authors:** Haidara Almansour, Saif Afat, Victor Fritz, Fritz Schick, Marcel Nachbar, Daniela Thorwarth, Daniel Zips, Arndt-Christian Müller, Konstantin Nikolaou, Ahmed E. Othman, Daniel Wegener

**Affiliations:** 1 Department of Diagnostic and Interventional Radiology, Eberhard-Karls University, 72076 Tuebingen, Germany; haidara.al-mansour@med.uni-tuebingen.de (H.A.); saif.afat@med.uni-tuebingen.de (S.A.); konstantin.nikolaou@med.uni-tuebingen.de (K.N.); 2 Section for Experimental Radiology, Department of Radiology, Eberhard-Karls University, 72076 Tuebingen, Germany; victor.fritz@med.uni-tuebingen.de (V.F.); fritz.schick@med.uni-tuebingen.de (F.S.); 3 Section for Biomedical Physics, Department of Radiation Oncology, Eberhard-Karls University, 72076 Tuebingen, Germany; marcel.nachbar@med.uni-tuebingen.de (M.N.); daniela.thorwarth@med.uni-tuebingen.de (D.T.); 4 German Cancer Consortium (DKTK), Partner Site Tuebingen and German Cancer Research Center (DKFZ), 69120 Heidelberg, Germany; daniel.zips@med.uni-tuebingen.de; 5 Department of Radiation Oncology, Eberhard-Karls University, 72076 Tuebingen, Germany; arndt-christian.mueller@med.uni-tuebingen.de (A.-C.M.); Daniel.wegener@med.uni-tuebingen.de (D.W.); 6 Department of Neuroradiology, University Medical Center Mainz, 55131 Mainz, Germany

**Keywords:** prostate carcinoma, mpMRI, adaptive radiotherapy, image guidance, MR-Linac, PIRADS, image quality, lesion detection

## Abstract

**Simple Summary:**

High-precision MR-guided radiotherapy (MRgRT) constitutes the state-of-the-art in the sphere of personalized prostate cancer treatment. To this end, integrating a 1.5 T scanner with a linear accelerator led to the development of MR-Linac (MRL), which could be considered a novel deflection point in radiation oncology. Since the success of both diagnosis and radiation treatment is highly dependent on image quality, geometric integrity, and lesion conspicuity, it is important to investigate the quality of these sequences in comparison to the current diagnostic gold standard multiparametric MRI at 3T (MRI_3T_), which has not been done before. The purpose of this study is to conduct a qualitative and a quantitative analysis of MRL-images at 1.5 T in patients undergoing MRgRT planning for prostate cancer. Results from this study pave the way for developing safer and more efficient planning workflows in patients with prostate cancer undergoing MR-guided radiotherapy.

**Abstract:**

The objective of this study is to conduct a qualitative and a quantitative image quality and lesion evaluation in patients undergoing MR-guided radiation therapy (MRgRT) for prostate cancer on a hybrid magnetic resonance imaging and linear accelerator system (MR-Linac or MRL) at 1.5 Tesla. This prospective study was approved by the institutional review board. A total of 13 consecutive patients with biopsy-confirmed prostate cancer and an indication for MRgRT were included. Prior to radiation therapy, each patient underwent an MR-examination on an MRL and on a standard MRI scanner at 3 Tesla (MRI_3T_). Three readers (two radiologists and a radiation oncologist) conducted an independent qualitative and quantitative analysis of T2-weighted (T2w) and diffusion-weighted images (DWI). Qualitative outcome measures were as follows: zonal anatomy, capsule demarcation, resolution, visibility of the seminal vesicles, geometric distortion, artifacts, overall image quality, lesion conspicuity, and diagnostic confidence. All ratings were performed on an ordinal 4-point Likert scale. Lesion conspicuity and diagnostic confidence were firstly analyzed only on MRL. Afterwards, these outcome parameters were analyzed in consensus with the MRI_3T_. Quantitative outcome measures were as follows: anteroposterior and right left diameter of the prostate, lesion size, PI-RADS score (Prostate Imaging—Reporting and Data System) and apparent diffusion coefficient (ADC) of the lesions. Intergroup comparisons were computed using the Wilcoxon-sign rank test and *t* tests. A post-hoc regression analysis was computed for lesion evaluation. Finally, inter-/intra-reader agreement was analyzed using the Fleiss kappa and intraclass correlation coefficient. For T2w images, the MRL showed good results across all quality criteria (median 3 and 4). Furthermore, there were no significant differences between MRL and MRI_3T_ regarding capsule demarcation or geometric distortion. For the DWI, the MRL performed significantly less than MRI_3T_ across most image quality criteria with a median ranging between 2 and 3. However, there were no significant differences between MRL and MRI_3T_ regarding geometric distortion. In terms of lesion conspicuity and diagnostic confidence, inter-reader agreement was fair for MRL alone (Kappa = 0.42) and good for MRL in consensus with MRI_3T_ (Kappa = 0.708). Thus, lesion conspicuity and diagnostic confidence could be significantly improved when reading MRL images in consensus with MRI_3T_ (Odds ratio: 9- to 11-fold for the T2w images and 5- to 8–fold for the DWI) (*p* < 0.001). For measures of lesion size, anterior-posterior and right-left prostate diameter, inter-reader and intersequence agreement were excellent (ICC > 0.90) and there were no significant differences between MRL and MRI_3T_ among all three readers. In terms of Prostate Imaging Reporting and Data System (PIRADS) scoring, no significant differences were observed between MRL and MRI_3T_. Finally, there was a significant positive linear relationship between lesion ADC measurements (r = 0.76, *p* < 0.01) between the ADC values measured on both systems. In conclusion, image quality for T2w was comparable and diagnostic even without administration of spasmolytic- or contrast agents, while DWI images did not reach diagnostic level and need to be optimized for further exploitation in the setting of MRgRT. Diagnostic confidence and lesion conspicuity were significantly improved by reading MRL in consensus with MRI_3T_ which would be advisable for a safe planning and treatment workflow. Finally, ADC measurements of lesions on both systems were comparable indicating that, lesion ADC as measured on the MRL could be used as a biomarker for evaluation of treatment response, similar to examinations using MRI_3T_.

## 1. Introduction

Magnetic resonance imaging (MRI) constitutes the cornerstone in the diagnosis and staging of prostate cancer as it has an indispensable role in the clinical routine due to its exquisite delineation of prostate and pelvic anatomy [1,2]. Recent technological developments have expanded the role of MRI and revolutionized personalized prostate cancer treatment via high-precision MR-guided radiotherapy (MRgRT). To this end, integrating a 1.5 T scanner with a linear accelerator led to the development of MR-Linac (MRL), which can be considered a novel deflection point in radiation oncology [3,4]. This integration builds on the idea that safe radiation therapy is predicated on well focused tumor treatment without markedly injuring the neighboring healthy tissues, since therapeutic radiation doses required to destroy cancerous lesions mostly exceed the tolerable threshold by healthy tissues [5].

On the MRI table, the novel MRL system enables a continuous “real-time” or “online” visualization of the prostate and neighboring organs with high soft-tissue contrast and allows for real-time plan adaptation and dynamic decision-making. The MRL T2-weighted (T2w) sequence of the MRL used for daily plan adaptation requires acquisition time of less than two minutes. Furthermore, the 1.5T MRL enables real-time functional imaging like diffusion-weighted-imaging (DWI) for response assessment and biological radiation adaptation. Since the success of both diagnosis and radiation treatment is highly dependent on image quality, geometric integrity and lesion conspicuity, it is important to investigate the quality of these sequences in comparison to the current diagnostic gold standard which is multiparametric MRI at 3T (MRI_3T_) [6].

Currently, MRI_3T_ serves as a diagnostic standard of reference for the images that are recorded on MR-Linac. Using the images recorded on MRI_3T_ aids physicians in radiation treatment planning, lesion segmentation as well as segmentation of the neighboring organs to protect the organs at risk (OAR).

The purpose of this study is to conduct a qualitative and a quantitative analysis of T2w and DW-images recorded on MRL at 1.5 T in patients undergoing MRgRT planning for prostate cancer using MRI_3T_ as a standard of reference.

## 2. Materials and Methods

### 2.1. Study Design and Patient Population

This prospective single-center study was approved by the institutional review board. All patients participated in the M-base Pro 1.0 or M-base HyPro 2.0 studies for primary radiation therapy of localized prostate cancers at our clinic for radiation oncology (ClinicalTrials.gov Identifiers: NCT02724670; NCT03880851). Within those studies, all patients consented in writing to undergo a second MRI examination at 3T. Between February 2019 and June 2020, a total of 13 consecutive patients with biopsy-confirmed prostate cancer and complete MRL and MRI_3T_ data sets were included.

### 2.2. MRI Technique and Acquisition Parameters

#### 2.2.1. MRI Examination at MRL

Peristalsis was not suppressed prior to the MRI examination and no contrast medium was administered. All examinations were performed in supine position in the hybrid 1.5 T MRI scanner (Elekta Unity™, Philips, Stockholm, Sweden), which is a modified 1.5T Philips Ingenia (Best, The Netherlands). An 8-channel body array coil was utilized for signal reception. The coil is designed to have minimal attenuation of the radiation beam by placing 4 channels anterior to the patient and 4 channels posterior to the patient. The posterior coil is embedded in the patient couch, while the anterior coil rests on a holder which prevents contact to the patient’s body [7]. No endorectal coils were applied. All patients underwent an MRgRT planning protocol consisting of axial T2w imaging in the axial plane and DWI using three different b-values (50 s/mm^2^, 500 s/mm^2^, and 800 s/mm^2^) followed by reconstruction of apparent diffusion coefficient (ADC) maps.

#### 2.2.2. MRI Examinations at 3T

Prior to the MRI examination, peristalsis was suppressed by the IV administration of 20 mg of butyl scopolamine (Buscopan, Boehringer, Germany). Body-weight adapted contrast agent was administered (0.1 mmol/kg gadobutrol; Gadovist, Bayer Healthcare, Leverkusen, Germany) with a flow rate of 1.5 mL/s followed by a saline flush of 20 mL using a power injector. All examinations were performed in supine position using a clinical 3T MRI scanner (MAGNETOM Skyra, Siemens Healthcare, Erlangen, Germany). A combined coil setup of an 18-channel body coil and 12 elements of a 32-channel spine coil was utilized. No endorectal coils were used. All included patients underwent a routine multi parametric (mpMRI) consisting of T2w imaging in three dimensions angulated to the prostate, as well as DWI using three different b-values (50 s/mm^2^, 500 s/mm^2^, 1000 s/mm^2^ and a calculated 2000 s/mm^2^) with reconstruction of ADC maps. ADC-maps were generated and analyzed offline using an in-house developed program based on MATLAB software (MATLAB; MathWorks, Natick, MA, USA) (Source Code S1). A standard log-linear model was applied to calculate ADC-values voxel-wise in each slice and for each patient for MRL and MRI_3T_. The acquisition parameters used for MRL and MRI_3T_ imaging are given in Table 1.

### 2.3. MR-Image Evaluation

In each patient, T2-weighted, DWI images, and ADC maps rom examinations on MRL and MRI_3T_ were independently presented to three readers in a random order (reader 1, a radiation oncologist with 6 years of experience in radiation oncology, reader 2, a radiologist with 3 years of experience in interpreting prostate MRI, reader 3, a radiologist with 6 years of experience in interpreting prostate MRI). In addition, dynamic contrast enhanced (DCE) images for MRI_3T_ were reviewed. A dedicated workstation (GE Healthcare Centricity™ PACS RA1000, Milwaukee, WI, USA) was utilized for image analysis.

#### 2.3.1. Qualitative Image Analysis

Qualitative ratings were performed on an ordinal 4-point Likert scale with 4 being the best: (1 = non-diagnostic; 2 = poor; 3 = good; 4 = excellent). “Zonal anatomy”: the ability to differentiate peripheral zone (PZ) from transition zone (TZ). “Resolution”: the ability to identify anatomical details within the prostate. “Capsule demarcation”: the ability to continuously identify the prostatic capsule. Overall image quality (1 = non-diagnostic; 2 = poor image quality, which merits the repetition of MRI examination; 3 = good image quality; 4 = excellent image quality). Diagnostic confidence (1 = non-diagnostic; 2 = poor confidence which merits the repetition of MRI examination; 3 = good confidence; 4 = very good confidence). Lesion conspicuity was subjectively rated as follows: (1 = delineation of lesion margins is not possible 2 = lesion margins are recognizable but blurry; 3 = good delineation of lesion margins good recognizable; 4 = excellent delineation of lesion margins) [8].

Geometric distortion and artifacts (wrapping, ghosting, susceptibility etc.) were rated separately as follows: Geometric distortion (1 = severe distortion 2 = moderate distortion; 3 = low distortion, 4 = no visible distortion). Artifacts (1 = abundant artifacts leading to image degradation; 2 = severe impediment of image quality by artifacts; 3 = slight impediment of image quality by artifacts, 4 = no visible artifacts).

#### 2.3.2. Quantitative Image Analysis

After qualitative image analysis, the three readers measured the size of the prostatic lesions on T2w and DW sequences in MRL and MRI_3T_ image sets. In addition, the Prostate Imaging Reporting and Data System (PIRADS) score for T2 and DWI image datasets for MRL and MRI_3T_ were evaluated. For assessment of representative ADC values, an elliptic region-of-interest (ROI) was placed by one reader within the lesions for each patient in MRL and MRI_3T_ image sets [9].

Geometric distortion, diameters in the anteroposterior (AP) and left-right (LR) diameter of the prostate were assessed on the level of the verumontanum on axial image sets of both MRL and MRI_3T_, respectively. The T2w images of MRI_3T_ served as the standard of reference regarding prostatic anatomic margins. Geometric distortion was defined as the differences in diameters between T2w on MRI_3T_ and DWI on MRL [9].

#### 2.3.3. Lesion Analysis on MRL with and without MRI_3T_

Lesion conspicuity and diagnostic confidence were first analyzed on MRL images only. Afterwards, these outcome parameters were analyzed again on the MRL images side by side in consensus with images recorded on the MRI_3T._ Differences between MRL ratings regarding the aforementioned outcome parameters were then computed.

### 2.4. Statistical Analysis 

Continuous variables were reported as mean and standard deviation or median and interquartile range according to data distribution. The Wilcoxon-signed rank test and paired *t*-test were used for intersequence comparisons. A post-hoc logistic regression analysis (generalized linear model for ordinal valuables) [10,11] was computed for lesion evaluation. Pearson correlation coefficient was used to test the correlation of ADC measurements between MRL and MRI_3T_. Fleiss Kappa was used to evaluate inter-reader agreement of categorical variables. Values from 0.0 to 0.2 indicate slight agreement, 0.21 to 0.40 fair agreement, 0.41 to 0.60 moderate agreement, 0.61 to 0.80 substantial agreement, and values ranging from 0.81 to 1.0 indicate almost perfect or perfect agreement [12]. Intraclass correlation coefficient (ICC) was used to compute inter-reader agreement for continuous variables. An ICC of less than 0.4 indicates poor agreement, of 0.40 to 0.59 fair agreement, of 0.60 to 0.74 good agreement, and an ICC of 0.75 to 1.00 indicates excellent agreement [13]. Bland–Altman plots were utilized to compare intra-rater agreement of prostate lesion size measurements for each reader. Level of significance was set at 0.05. Statistical analyses were performed using SPSS (v26.0, IBM-Corp, Armonk, NY, USA).

## 3. Results

**Figure 1:**
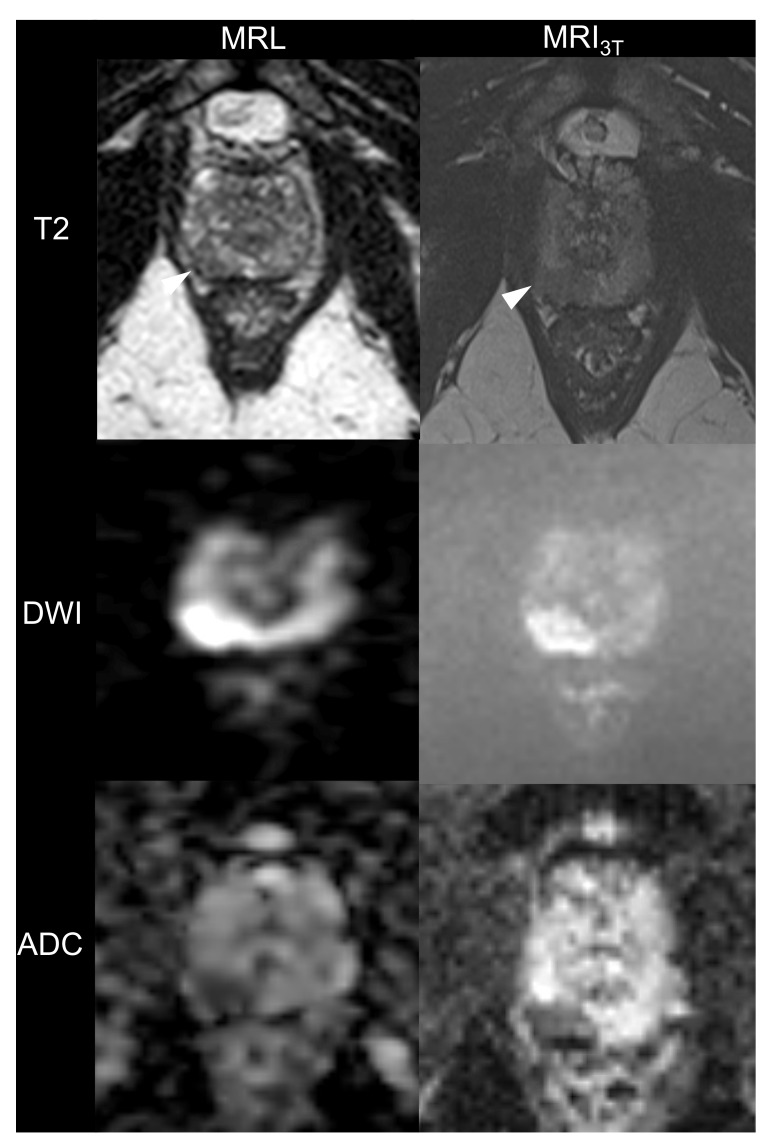
60-year-old man with prostate-specific antigen level of 2.28 ng/mL and biopsy-confirmed prostate cancer. Axial T2-weighted images, diffusion-weighted images (DWI) and apparent diffusion coefficient (ADC) maps acquired with MR-Linac (MRL) operating at 1.5 T and corresponding images acquired on a standard 3T MRI (MRI_3T_). Note the hypointense region in the apical aspect of the right peripheral zone (arrows). Images generated with the MRI_3T_ have higher overall image quality, especially in the DWI. Histology of specimen yielded stage cT2a tumor.

**Figure 2:**
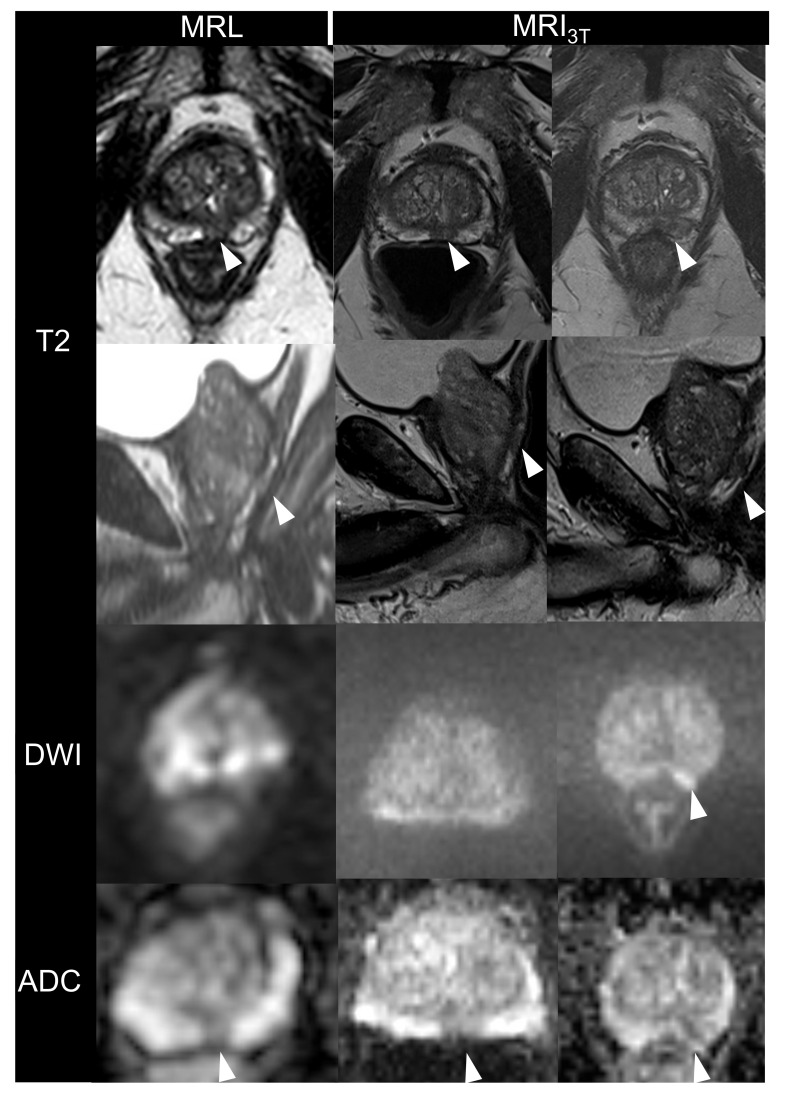
73-year-old man with an initial prostate-specific antigen level of 7.30 ng/mL and biopScheme 2. weighted images, diffusion-weighted images (DWI) and apparent diffusion coefficient (ADC) maps acquired by the MR-Linac (MRL) at 1.5 T and corresponding images from standard MRI at 3T (MRI_3T_). The left and middle column show MRL and MRI_3T_ images after 3 months of androgenic suppression therapy. The right column illustrates MRI_3T_ images before initiation of androgenic suppression therapy. Note that the lesion in the DWI before initiation of hormone therapy is slightly hyper-intense (arrow), whereas after hormonal therapy, the lesion appears rather iso-intense on DWI. Note the hypo-intense region in the apical posterior-medial aspect of the left peripheral zone (arrows). Again, images generated with the MRI3T have better overall image quality when compared to MRL especially in the DWI. Histology of specimen yielded stage cT2c tumor.

**Figure 3:**
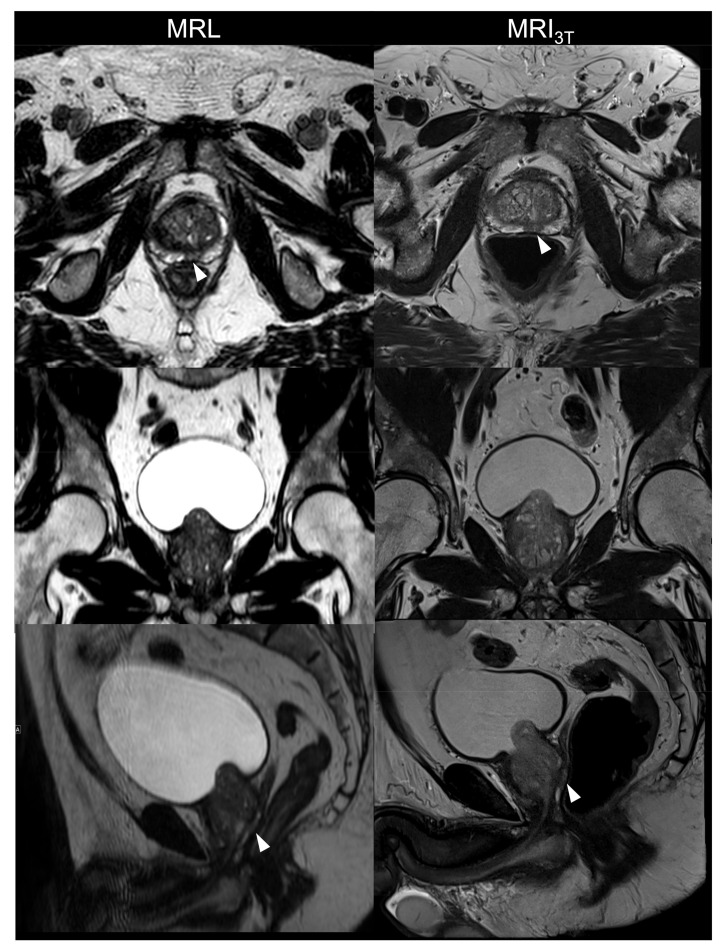
Illustration of T2-weighted axial (**first row**), coronal (**second row**) and sagittal (**third row**) images acquired using MR-Linac (MRL) at 1.5 T and corresponding images recorded on a standard MRI at 3 T (MRI_3T_) of the same 73-year-old male as in Figure 2. Diffusion-weighted images (DWI) and apparent diffusion coefficient (ADC) maps are depicted. The cancerous lesion on the apical posterior-medial aspect of the left peripheral zone is shown on the axial and sagittal images (arrows).

A total of 13 males aged 69 ± 7 years with biopsy-confirmed prostate cancer were included. Patient characteristics are given in Table 2. All imaging studies were successfully performed and evaluated. Figure 1, Figure 2 and Figure 3 are illustrations of T2w and DWI sequences as well as ADC maps for three patients derived from examinations on MRL and MRI_3T_.

### 3.1. Qualitative Image Analysis

#### 3.1.1. Image Quality

Inter-reader agreement for image quality parameters was good (according to the given grading) for both, MRL (Kappa = 0.610) and MRI_3T_ (Kappa = 0.721). Detailed results for the three readers are given in Table 3. In summary, the MRL showed good results for the T2w across all image quality criteria (median 3 and 4), however significantly inferior to the MRI_3T_. Furthermore, there were no significant differences between MRL and MRI_3T_ regarding capsule demarcation or geometric distortion. For the DWI, the MRL performed significantly less than MRI_3T_ across most image quality criteria with a median ranging between 2 and 3. Finally, there were no significant differences between MRL and MRI_3T_ regarding geometric distortion.

#### 3.1.2. Lesion Conspicuity and Diagnostic Confidence on MRL Alone and in Consensus with MRI_3T_

Inter-reader agreement for lesion conspicuity and diagnostic confidence was fair for MRL alone (Kappa = 0.42) and good for MRL in consensus with MRI_3T_ (Kappa = 0.708). Detailed results for the three readers are given in Table 4. For MRL alone, lesion conspicuity and diagnostic confidence were good in T2w (median 3) but poor in DWI (median 1 to 2). There was a significant improvement in lesion conspicuity and diagnostic confidence when MRL images were viewed in consensus with MRI_3T_ (Very good, median 4 in T2W and good, median 3 in DWI) (Table 4). Furthermore, a post-hoc logistic regression analysis (generalized linear model for ordinal valuables) [10,11] was computed to investigate whether reading MRL images in consensus with MRI_3T_ played a role in improving lesion conspicuity and diagnostic confidence. For every 1-point increase in lesion conspicuity, the estimated odds ratio revealed a positive relation with “MRL with MRI_3T_” of nearly 9-fold for the T2w images and of nearly 8-fold for the DWI in comparison to “MRL without MRI_3T_” (*p* < 0.001) (Table 5).

For every 1-point increase in diagnostic confidence, the estimated odds ratio revealed a positive relation with “MRL with MRI3T” of nearly 11-fold for the T2w images and of nearly 5-fold for the DWI in comparison to “MRL without MRI_3T_” (*p* < 0.001) (Table 5).

### 3.2. Quantitative Analysis 

#### 3.2.1. Lesion Size, Prostate Diameter and Geometric Distortion

Inter-reader agreement was excellent for lesion size, anterior-posterior and right-left prostate diameter (ICC > 0.92). Intersequence agreement (between MRL and MRI_3T_) for each reader was also excellent. There were no significant differences between MRL and MRI_3T_ regrading lesion size, anterior-posterior and right-left prostate diameters among all three readers (all *p* > 0.05). Details are given in Table 6. In terms of geometric distortion, for all three readers, there was no statistically significant difference between DWI images on MRL in comparison to T2w on MRI_3T_ (all *p* > 0.05) with the highest geometric distortion being 1.7 mm.

#### 3.2.2. PIRADS Scoring

Inter-reader agreement was substantial for both T2w (kappa = 0.803) and DWI (kappa = 0.770). The intersequence agreement ranged from (Kappa of 0.65 to 1) (Table 7). There were no significant differences regarding PIRADS scoring on T2 or DWI between MRL and MRI_3T_.

#### 3.2.3. Lesion ADC Measurements

For MRL, the mean ADC values showed a slightly higher trend with a mean of 1.07 × 10^−3^ mm^2^/s ± 0.4 × 10^−3^ mm^2^/s. For MRI_3T,_ the mean ADC value was 0.8 × 10^−3^ mm^2^/s ± 0.2 × 10^−3^ mm^2^/s. There was no statistically significant difference between MRL and MRI_3T_. Pearson correlation coefficient revealed a positive linear correlation for lesion ADC measurements (*r* = 0.76, *p* < 0.01).

## 4. Discussion

This prospective study is the first clinical comparison of MR-Linac at 1.5 T to a diagnostic scanner at 3 T in the context of planning MRgRT of prostate cancer. This study shows that comparable quality can be achieved in clinical routine using a fast T2w sequence. Furthermore, we could demonstrate that the weaker magnetic field strength does not significantly compromise PIRADS scoring or lesion size measurements and that the MRL system offers a high geometric integrity. Simultaneously, we showed that functional imaging using DWI needs optimization as it did not reach a high diagnostic level.

The MRL “fast” T2-sequence (with acquisition time less than 2 min) provides sufficient soft tissue contrast for daily plan adaptation and is fine-tuned for speedy and robust image acquisition during radiation treatment as elapsed time is a risk factor for patient motion and organ motion (bladder filling, changes in anorectal filling, and target volume motion) [14,15]. This sequence is the gold standard for daily RT planning on the MRL [14] and used in all “Unity” sites. This allows sharing of data and building of models. As expected the quality of the fast T2w MRL sequence was inferior to a MRI_3T_ providing high contrast and resolution as well as low motion artifacts. Here, high image quality is further facilitated by the use of contrast agents and spasmolytica (Table 3). Inter-reader reliability was high, further highlighting the good comparability of this modality. In addition, when comparing T2w imaging on MRL with or without addition of the image data from MRI_3T_, the logistic regression model revealed a 9-fold increase in diagnostic confidence for the combination. Therefore, it is recommended to combine MRL prostate image viewing and interpretation with results from recent diagnostic MR examinations.

DWI constitutes a fundamental part of mpMRI of the prostate and has shown its value in detecting clinically relevant prostate cancer as well as in assessing cancer aggressiveness [8,16,17]. However, this sequence is challenged by excessive artifacts and geometric distortion as well as susceptibility to B0-field inhomogeneity [8,16,17]. A major goal of a 1.5T MRL is to exploit having access to functional imaging in real-time which would possibly allow real-time response- or toxicity assessment during radiation treatment and subsequent immediate reaction and plan adaptation [18]. Therefore, this sequence is also tuned for short acquisition time and a certain inferiority to a state-of-the art MRI_3T_ was expected. Neither artefacts nor geometric distortion impeded image quality significantly. This might be explained by the fact that none of the included patients had metallic implants. However, our findings show that the current MRL-DWI images did not reach high diagnostic quality (zonal anatomy 2/4, resolution 2/4, overall image quality 2/4, see Table 3). Using the current settings, lesion conspicuity and diagnostic confidence were not sufficient for reliable interpretation in this cohort according to the three readers. The suboptimal DWI images could be explained by the fact that the MRL-DWI images are currently not being routinely used for treatment planning or plan adaptation. These images were acquired on the first commercial MRL system with a self-made protocol for scientific purposes. The large FoV was deliberately chosen with the aim of allowing response- and toxicity assessment not only of the prostate but of rectal wall and adjacent bladder as well as these being the most relevant and dose-limiting organs at risk. However, this might have contributed to the inferior quality of the DWI for the prostate as size of FoV was demonstrated to influence image quality on 3T scanners [19] and on the MRL [20]. In addition, MpMRI is usually conducted with the aid of spasmolytic agents (i.e., butyl scopolamine), contrast agent and with fine-tuned sequences and specifications while images on the MRL are acquired daily for several weeks along fractionated radiotherapy and the repeated, potentially harmful use of contrast agent or butyl scopolamine is not justified on a daily basis.

DWI on MRL also profited in the linear regression model from consulting the MRI_3T_ (odds ratio increases for lesion conspicuity of about 8-fold, for diagnostic confidence 5-fold), so that we advise the use of a corresponding MRI_3T_ as a reference standard.

Using the DWI-derived apparent diffusion coefficient values (ADC) of prostatic lesions as recorded on MRI_3T_ a standard of reference, lesion ADC values on MRL were comparable. This indicates that ADC on MRL may serve as a quantitative prognostic imaging biomarker similarly to MRI_3T_. Future studies may use ADC on MRL to assess treatment response and to predict clinical outcome. Indeed, this merits an independent prospective examination.

In addition, our data demonstrate low geometric distortion for DWI on the MRL. This is important because geometric integrity is crucial in the setting of radiation treatment. Therefore, MRL sequences need high readout gradient bandwidths at the cost of signal to noise ratio [21]. In comparison with diagnostic MRIs, the latter should also be set for geometric accuracy and should use spatial correction techniques. Both lesion size and AP and RL diameter of the prostate were comparable between modalities as well as readers (Table 6). This finding is in accordance with a phantom MRL study by Wang et al. [7], which showed a distortion of under 0.5 mm and a “first-in-man” study by Raaymakers et al. [22] in four patients with lumbar metastases, showing a distortion of between 0.2 and 0.4 mm. This underlines a good compensation and low impact of both system-specific and patient-specific causes of distortion [23,24] on the MRL images in our cohort.

Previous studies made a comparison between a standard diagnostic 1.5 T and 3 T prostate MRI [2,25]. For instance, Ullrich et al. [25] showed that both field strengths had similar diagnostic performance and PIRADS scoring with an excellent inter-reader agreement for image quality and PIRADS score. In our cohort, PIRADS scoring was similar on MRL and MRI_3T_ with a substantial inter-reader reliability.

The findings of this study should be interpreted within the context of its limitations. First, there is the small patient population who underwent prostate imaging at both devices. It is a fact that the strain associated with a second MRI examination is not well tolerated by all patients, so we included 13 consecutive patients during an 18-month period. Second, acquisition parameters of the MRL were different from the 3T MRI scanner and did not conform to the PIRADS technical recommendations, inherently creating comparison challenges. However, the main purpose of the MRL is to acquire a quick and reliable image for radiation planning and decision-making in real-time and not make the diagnosis of prostate cancer in a suspected lesion. Thirdly, seven out of thirteen-patients in this study had undergone neoadjuvant hormone- ablative treatment prior to the examinations. This study deliberately focused on the clinical comparability “regardless” of those technical or oncological differences as that is what clinical routine demands of any two modalities. The endpoints of this study remain relevant in spite of those distinctions.

## 5. Conclusions

In conclusion, image quality of T2w imaging was comparable and diagnostic even without administration of spasmolytic- or contrast agents, while DWI images did not reach a sufficient diagnostic level and need to be optimized for further exploitation of real-time functional imaging in the setting of MRgRT. Both for T2w and DWI imaging, lesion conspicuity and diagnostic confidence were significantly improved after reading MRL in consensus with MRI_3T_ which would be advisable for a safe planning and treatment workflow. Finally, ADC measurements were comparable, which indicates that lesion ADC as measured on the MRL might be used as a biomarker for evaluation of treatment response, similar to common procedures using MRI_3T_.

## Figures and Tables

**Table 1:** Magnetic Resonance Acquisition Parameters of T2-weighted images and DWI for MRL and MRI_3T._

Parameter	MR-Linac		MRI_3T_	
	3D T2	DWI-SPAIR	T2—TSE	DWI
Field of view (mm)	400 × 400	430 × 430	200 × 200	256 × 192
Acquisition voxel size (mm^3^)	1.5 × 1.5 × 2	3 × 3 × 4.5	0.65 × 0.52 × 3	1.67 × 1.33 × 4
Repetition time (msec)	1535	8907	4000–8000	5930
Echo time (msec)	278	77	90	76
Refocusing flip angle (degrees)	100	180	120	180
Section thickness (mm)	2	4.5	3	4
Time for acquisition (min:sec)	01:57	05:30	3:40–5:00	5:00–06:00
Bandwidth	740.3 Hz/Pixel	1902 Hz/Pixel	200 Hz/Pixel	1976 Hz/Pixel

3D: three-dimensional; T2 = T2-weighted; TSE: turbo spin echo; DWI: Diffusion-weighted Imaging; SPAIR: Spectral attenuated inversion recovery.

**Table 2:** Characteristics of the included patients.

Characteristics	Values (mean ± SD, (range))
Age (years)	69 ± 7 (59–79)
Prostate-specific antigen (ng/mL)	8.95 ± 4.95 (2.28–20.60)
Prostate volume (cm^3^)	40.8 ± 20.6 (14–83)
**Gleason score**	**Number of MRI examinations (*n* = 13)**
• 6	4
• 7a	5
• 7b	3
• 8	1
**Prostate cancer stage**	**Number of MRI examinations (*n* = 13)**
• c T2a	3
• c T2b	2
• c T2c	8
Patients who underwent androgen suppression therapy	7/13

SD = standard deviation.

**Table 3:** Comparison of qualitative image criteria between MR-Linac (MRL) and standard MRI at 3T (MRI_3T_) for T2w and DWI sequences.

Outcome Parameters	T2w_MRL_	T2w _MRI-3T_	*p* Value *	DWI_MRL_	DWI_MRI-3T_	*p* Value *
	Reader 1	Reader 1	Reader 1	Reader 1	Reader 1	Reader 1
	Reader 2	Reader 2	Reader 2	Reader 2	Reader 2	Reader 2
	Reader 3	Reader 3	Reader 3	Reader 3	Reader 3	Reader 3
Zonal anatomy	3 (0)	4 (0)	**0.002**	2 (1)	4 (1)	**0.003**
	3 (1)	4 (0)	**0.001**	2(0)	4 (0)	**0.001**
	3 (0)	4 (0)	**0.001**	2 (1)	4 (0)	**0.001**
Resolution	3 (1)	4 (0)	**0.003**	2 (0)	4 (0)	**0.001**
	3 (1)	4 (0)	**0.001**	2 (0)	4 (0)	**<0.001**
	3 (0)	4 (0)	**<0.001**	2 (0)	4 (0)	**0.001**
Capsule demarcation	4 (0)	4 (0)	0.157	3 (1)	4 (1)	**0.01**
	4 (0)	4 (0)	0.317	3 (1)	4 (1)	**0.002**
	4 (1)	4 (0)	0.083	3 (1)	4 (1)	**0.004**
Visibility of seminal vesicles	4 (1)	4 (0)	0.083	2 (1)	4 (0)	**0.002**
	3(1)	4(0)	**0.001**	2 (1)	4 (0)	**0.001**
	3(0)	4(0)	**<0.001**	3 (1)	4 (0)	**0.001**
Artifacts	3 (0)	4 (0)	**0.001**	3 (1)	4 (0)	**0.01**
	3 (1)	4 (0)	**0.001**	3 (1)	4 (0)	**0.004**
	3 (1)	4 (0)	**0.001**	3 (0)	4 (0)	**0.002**
Geometric distortion	4 (1)	4 (0)	0.157	4 (0)	4 (0)	0.317
	4 (0)	4 (0)	0.157	4 (0)	4 (0)	0.157
	4 (1)	4 (1)	0.655	4 (0)	4 (0)	0.083
Overall image quality	3 (0)	4 (0)	**0.001**	2 (1)	4 (0)	**0.002**
	3 (1)	4 (0)	**0.001**	2 (1)	4 (0)	**0.002**
	3 (0)	4 (0)	**< 0.001**	2 (1)	4 (0)	**0.002**

Data is given as median (Interquartile range); MRL: MR-Linac; MRI_3T_: Standard MRI at 3 Tesla.T2w = T2-weighted; DWI: Diffusion-weighted Imaging. * Bold denotes statistical significance.

**Table 4:** Comparison of lesion conspicuity and diagnostic confidence on MR-Linac (MRL) alone and on MRL in consensus with the MRI_3T_ (MRL with MRI_3T_).

Outcome Parameters	T2w_MRL_	T2w _MRL_ with T2_MRI-3T_	*p* Value *	DWI_MRL_	DWI_MRL_ with DWI_MRI-3T_	*p* Value
	Reader 1	Reader 1	Reader 1	Reader 1	Reader 1	Reader 1
	Reader 2	Reader 2	Reader 2	Reader 2	Reader 2	Reader 2
	Reader 3	Reader 3	Reader 3	Reader 3	Reader 3	Reader 3
Lesion Conspicuity	3 (1)	4 (1)	**0.014**	2 (3)	3 (2)	**0.023**
	3 (1)	3 (1)	**0.004**	1 (1)	3 (1)	**0.004**
	3 (1)	4 (1)	**0.002**	2 (1)	3 (1)	**0.006**
Diagnostic Confidence	3 (1)	4 (1)	**0.004**	2 (3)	3 (2)	**0.011**
	3 (1)	4 (1)	**0.005**	2 (1)	3 (1)	**0.004**
	3 (1)	4 (1)	**0.001**	3 (1)	3 (1)	**0.003**

Data is given as median (Interquartile range); MRL: MR-Linac; MRI_3T_: Standard MRI at 3 Tesla. T2w = T2-weighted; DWI: Diffusion-weighted Imaging. * Bold denotes statistical significance.

**Table 5:** Post-hoc logistic regression results for lesion conspicuity and diagnostic confidence in terms of reading the MRL images without MRI3T or in consensus with MRI3T.

Outcome Parameters	Likelihood Ratio-Chi-Square	Estimate (B)	SE	Wald-Chi-Square	*p* Value	Odds Ratio Exp(B)	95%CI
Lesion conspicuity T2w	22.271	2.197	0.516	18.117	*p* < 0.001	8.997	(3.272–24.742)
Lesion conspicuity DWI	25.528	2.063	0.475	18.836	*p* < 0.001	7.870	(3.1–19.98)
Diagnostic confidence T2w	26.987	2.445	0.522	21.935	*p* < 0.001	11.527	(4.144–32.065)
Diagnostic confidence DWI	16.379	1.617	0.453	12.728	*p* < 0.001	5.036	(2.072–12.239)

T2w: T2-weighted; DWI: Diffusion-weighted imaging; B: Regression co-efficient; SE: Standard error; CI: Confidence Interval; Exp(B): odds ratio based on the exponentiation of the B coefficient.

**Table 6:** Comparison of lesion size measurement as well as prostate diameter measurements on T2w- and DWI between MRL and as well intersequence agreement between MRL and MRl_3T_ for each reader.

Reader 1								
	T2_MRL_	T2_MRI-3T_	*p* Value	ICC *	DWI_MRL_	DWI _MRI-3T_	*p* Value	ICC *
Lesion size (mm)	13.4 ± 4.7	13.2 ± 4.6	0.48	0.98	13.5 ± 4.6	13 ± 4.8	0.13	0.98
AP Diameter	36 ± 8.4	35.8 ± 8.4	0.61	0.99	35.9 ± 8.5	35.2 ± 9.1	0.25	0.98
RL Diameter	46.7 ± 6.6	46.6 ± 7.2	0.85	0.98	47.9 ± 7.2	46.1 ± 8.6	0.17	0.91
**Reader 2**								
Lesion size (mm)	13 ± 5.1	12.7 ± 4.9	0.41	0.98	12.9 ± 4.9	13.1 ± 4.8	0.27	0.99
AP Diameter	35.7 ± 7.9	35.8 ± 7.2	0.84	0.99	36.08 ± 8.9	36 ± 9.1	0.85	0.99
RL Diameter	46.3 ± 6.6	46.5 ± 7.3	0.78	0.98	47.9 ± 7.1	46.7 ± 8.1	0.33	0.92
**Reader 3**								
Lesion size (mm)	13.1 ± 4.2	13.4 ± 5	0.55	0.95	12.8 ± 5.2	13 ± 4.9	0.65	0.98
AP Diameter	36.1 ± 8.5	36 ± 8.5	0.73	0.99	35.9 ± 9	36.08 ± 8.9	0.74	0.99
RL Diameter	46.3 ± 6.4	46.2 ± 7.1	0.72	0.98	47.9 ± 7.7	46.6 ± 8.7	0.31	0.91

Data is given as mean ± standard deviation. * ICC: Intraclass correlation coefficient refers to intersequence agreement for each reader. AP: anterior-posterior; RL: right-left; ADC: apparent diffusion coefficient; PZ: peripheral zone; TZ: transition zone; MRL: MR-Linac; MRI-3T: standard MRI at 3 Tesla; T2: T2-weighted; DWI: Diffusion-weighted imaging.

**Table 7:** Comparison of T2w- and DWI- PIRADS score between MRL and MRI3T for each reader as well intersequence agreement between MRL and MRl3T for each reader.

PIRADS	Reader 1		Reader 2		Reader 3	
T2-Score	T2_MRL_	T2_MRI-3T_	T2_MRL_	T2_MRI-3T_	T2_MRL_	T2_MRI-3T_
0	0	0	0	0	0	0
1	0	0	0	0	0	0
3	1	1	1	0	0	0
4	7	7	8	9	8	8
5	5	5	4	4	5	5
*p* value	1		0.317		1	
Inter-sequence agreement	1		0.840		1	
**DWI-Score**	**DWI_MRL_**	**DWI_MRI-3T_**	**DWI_MRL_**	**DWI_MRI-3T_**	**DWI_MRL_**	**DWI_MRI-3T_**
0	0	0	0	0	0	0
1	2	1	2	1	2	1
3	1	2	1	1	1	2
4	6	5	7	6	4	5
5	4	5	3	5	6	5
*p* value	0.157		0.102		1	
Inter-sequence agreement	0.774		0.64		0.774	

PIRADS: Prostate Imaging Reporting and Data System; MRL:MR-Linac; MRI-3T: standard MRI at 3 Tesla; T2w: T2-weighted; DWI: Diffusion-weighted imaging.

## Data Availability

The datasets used and analyzed during the current study are available from the corresponding author on reasonable request.

## Supplementary Materials

The following are available online at https://www.mdpi.com/article/10.3390/cancers13071533/s1, Source Code S1. Source code for the in-house developed program based on MATLAB software for ADC-mapping.
