# Supplementary material for: Prospective Image Quality and Lesion Assessment in the Setting of MR-Guided Radiation Therapy of Prostate Cancer on an MR-Linac at 1.5 T: A Comparison to a Standard 3 T MRI
Source: Cancers (Basel). 2021 Mar 26;13(7):1533. doi: 10.3390/cancers13071533 (PMC8036991; doi:10.3390/cancers13071533)
Supplement: Supplementary file 1 [file cancers-13-01533-s001.pdf]

Supplementary Material: Prospective Image Quality and Lesion Assessment in the Setting of MR-Guided Radiation Therapy of Prostate Cancer on an MR-Linac at 1.5 T: A Comparison to a Standard 3 T MRI

Haidara Almansour, Saif Afat, Victor Fritz, Fritz Schick, Marcel Nachbar, Daniela Thorwarth, Daniel Zips, Arndt-Christian Müller, Konstantin Nikolaou, Ahmed E Othman and Daniel Wegener

Source Code S1. Source code for the in-house developed program based on MATLAB software for ADC-mapping.

```
S
% Load dicom images
[fname, pname] = uigetfile( ...
    {   '*.*',    'All Files (*.*)'; ...
        '*.dcm','DICOM-files (*.dcm)'; ...
        '*.nema','NEMA-files (*.nema)'}, ...
        'Open Image Files');
fname = lower(fname);
cd(pname)
names=dir;
info=dicominfo(fname);
C = info.Columns;
R = info.Rows;
lin = double(R);
col = double(C);
tot_count = 0;
wait=waitbar(0, 'Data loading in progress, please wait ...');
for n = 1:(length(names))
    if (names(n).isdir)
        continue;
    end;
        tot_count = tot_count + 1;
    try
        info=dicominfo(names(n).name);
        data_tmp(:,:,tot_count)=dicomread(info);
        data(:,:,tot_count) = double(data_tmp(:,:,tot_count));
        slice_position(tot_count) = info.SliceLocation;
        BValue(tot_count) = info.DiffusionBValue;
    catch
        tot_count=tot_count-1;
    end
    waitbar(n/length(names));
end
close(wait);
slice_position_0 = min(slice_position);
bval = length((slice_position(slice_position == slice_position_0)));
sli = tot_count/bval;
for ibv = 1:bval
    bvalue(ibv) = BValue(ibv);
end
ima = reshape(data, lin, col, bval, sli);
```

```
% ADC-mapping

ADC_map = zeros(lin, col, sli);
xdata= bvalue;
wait1=timebar('Computation in progress...','Wait...');
for k=1:size(ima,4);
    for i=1:size(ima,1);
        for j=1:size(ima,2);
            if(ima(i,j,bval,k) > 0
                for m=1:bval
                    ydata(m) = log(ima(i,j,m,k));
                end
                calc_par = polyfit(xdata, ydata, 1);
                ADC_map(i,j,k) = -calc_par(1);
            end
        end
        timebar(wait1,((k-1)*lin+i)/(size(ima,4)*size(ima,1)));
    end
end
close(wait1);
% save data...
```
